# Recruiting Latino young adults into a vaping cessation study via social media: Feasibility and cost analysis

**DOI:** 10.18332/tpc/186146

**Published:** 2024-04-09

**Authors:** Rafael H. Orfin, Victoria Uceda, Cody Gardner, Brianna Estrada, Edward Tamayo, Ruthmarie Hernández-Torres, Dongmei Li, Irfan Rahman, Scott McIntosh, Deborah J. Ossip, Ana Paula Cupertino, Francisco Cartujano-Barrera

**Affiliations:** 1 Department of Public Health Sciences, University of Rochester Medical Center, Rochester, United States; 2 School of Medicine, St. George's University, United States; 3 Clinical and Translational Science Institute, University of Rochester Medical Center, Rochester, United States; 4 The Kick Vaping Latino Advisory Board; 5 Department of Psychiatry and Behavioral Sciences, Memorial Sloan Kettering Cancer Center, United States; 6 Department of Environmental Medicine, University of Rochester Medical Center, Rochester, United States; 7 Department of Surgery, University of Rochester Medical Center, Rochester, United States

**Keywords:** social media, vaping, recruitment, Latinos, young adults, vaping cessation

## Abstract

**INTRODUCTION:**

This study aims to assess the feasibility and cost of recruiting young Latino adults (aged 18–25 years) to participate in a vaping cessation study via social media and to describe the baseline characteristics of participants enrolled via social media.

**METHODS:**

Paid advertisements were launched using the Meta Ads platform, which serves ads to users on Facebook and Instagram. Key measures of audience targeting included ages 18–25 years, all genders, and the following interests: ‘electronic cigarettes’, ‘vape’, ‘Latin pop’, and ‘Latin music’. The advertisements invited young Latino adults to join a text messaging vaping cessation study. By clicking on the advertisements, interested individuals were directed to a website to fill in a contact form. The study team contacted individuals who filled in the form, assessed them for study eligibility, and, if eligible, enrolled them in the study.

**RESULTS:**

A total of 164 individuals completed the contact form, and 26 were successfully enrolled in the study. The enrollment efficiency ratio was 15.9% (26/164). The cost per enrollment was US$94.14. The participants’ mean age was 22.7 years (SD=1.6). Half of the participants (50%) were male, 38.5% were female, and 11.5% were gender non-conforming/non-binary. Two-thirds of the participants (69.2%) were born in the US, 23.1% in Puerto Rico, and 7.7% in Mexico. Eight participants (30.7%) selected Spanish as their language of preference. In terms of the type of vaping device, 16 participants (61.5%) indicated using disposables, 6 (23.1%) cartridges/pods, and 4 (15.4%) tanks/refillable. Sixteen participants (61.5%) reported using marijuana in e-cigarettes. Six participants (23.1%) had high e-cigarette dependence. Twenty participants (76.9%) had attempted to quit e-cigarettes in the past year.

**CONCLUSIONS:**

It is feasible to recruit young Latino adults for a vaping cessation study via social media. Social media offers a relatively low-cost approach to recruiting a diverse sample of Latino young adults who vape.

## INTRODUCTION

Electronic cigarette (e-cigarette) use (vaping) is a significant public health concern due to its associated health risks, including co-use of marijuana and alcohol, heavy metal exposure, and lung injury^1-4^. In response, research on vaping cessation is emerging^5-6^. However, emerging research in the US is hampered by limited racial and ethnic diversity among participants. For example, a recently completed randomized controlled trial (RCT) with young adults (aged 18–24 years) demonstrated the effectiveness of the first vaping cessation intervention^6^. Limitations of the RCT included the small representation of Latinos (10.6%) – the largest minority group in the US^7^ – and the exclusion of Spanish-speaking individuals^6^. Given the substantial gap in vaping control research among Latinos, the purpose of the present study was thus two-fold: 1) assess the feasibility and cost of recruiting Latino young adults (aged 18–25 years) into a vaping cessation study via social media, and 2) describe the baseline characteristics of participants enrolled via social media. Recruitment via social media was selected, given their high popularity among young adults^8^.

## METHODS

This is a secondary data analysis of the social media recruitment for the Kick Vaping study. Kick Vaping is a single-arm pilot study assessing the feasibility and acceptability of a vaping cessation text messaging intervention for Latino young adults^9^. A community advisory board of Latino young adults guided the implementation of the Kick Vaping study. Study procedures were approved and monitored by the University of Rochester Medical Center (URMC) Institutional Review Board (protocol number STUDY00007630).

**Figure 1:**
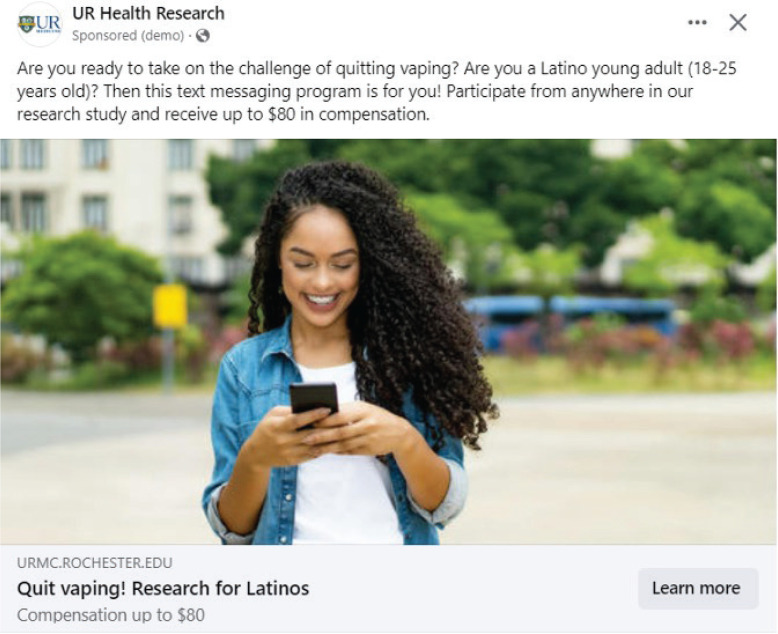
Advertisement inviting Latino young adults to join the vaping cessation study

Between March and November 2023, three paid advertisements (one in English, one in Spanish exclusive to the United States, and one in Spanish exclusive to Puerto Rico) were launched using the Meta Ads platform, which serves ads to users on Facebook and Instagram. Key elements of audience targeting included ages 18–25 years, all genders, and the following interests: ‘electronic cigarettes’, ‘vape’, ‘Latin pop’, and ‘Latin music’. The advertisements invited Latino young adults to join a vaping cessation study by clicking on it. The advertisements included different images of Latino male and female young adults; images did not include e-cigarettes. The advertisements also stated that participants could earn up to $80 for being part of the study (Figure 1). Participant compensation modeled previous tobacco cessation studies with the Latino community^10,11^. Interested individuals were directed to a website to fill in a contact form by clicking on the advertisements. The contact form, available in English and Spanish, collected the name, phone number, email, and preferred time to be contacted. The study team contacted individuals who filled in the form via phone and assessed them for study eligibility.

Individuals were considered eligible if they: 1) self-identified as Hispanic and/or Latino; 2) could read and speak English and/or Spanish; 3) were aged 18–25 years; 4) used electronic cigarettes at least one day per week within a typical week; 5) were interested in quitting vaping in the next 30 days; 6) had an active cellphone with unlimited text messaging capability; and 7) were willing to complete two study visits, over Zoom^®^ or a phone call, at baseline and at 12 weeks. Exclusion criteria were: 1) having used any tobacco products other than electronic cigarettes in the past seven days (including traditional cigarettes), 2) having a household member enrolled in the study, and 3) lived outside the US.

Eligible individuals were scheduled for a Zoom^®^ or phone call appointment. During the appointment, staff guided individuals through the process of written informed consent. The eligibility assessment and consent process were hosted in REDCap and are available in English and Spanish. Ineligible individuals were referred to the Wilmot Tobacco Cessation Center at the URMC if they were currently smoking cigarettes, and/or to the ‘This is Quitting’ program by the Truth initiative if they were currently vaping.

The baseline survey collected sociodemographic variables, including age, gender, sexual orientation, education level, marital status, and employment status. The survey also collected data on country of birth and language of preference. Vaping-related variables collected included type of vaping device (i.e. disposables, cartridges/pods, or tanks/refillable), use of marijuana in e-cigarettes, e-cigarette dependence, and if they made a quit attempt in the previous year. E-cigarette dependence was measured by the Penn State E-cigarette Dependence Index (PSECDI)^12^. The PSECDI is a 10-item scale that assesses dependence through the frequency of use, time to first e-cigarette use of the day, waking at night to use, perceived difficulty quitting, cravings, urges, and withdrawal^12^. PSECDI scores range from 0 to 20, with higher scores indicating greater e-cigarette dependence^12^. The baseline assessment was hosted in REDCap and available in English and Spanish.

Feasibility and cost were assessed via the enrollment efficiency ratio and cost per enrollment. Enrollment efficiency was the ratio of the number of individuals enrolled to the number who completed the contact form. Cost per enrollment was the total amount spent on the advertisements divided by the number of individuals enrolled. For the baseline characteristics, frequencies and percentages were calculated for categorical variables and means and standard deviations for continuous variables.

## RESULTS

A total of 164 individuals completed the contact form. Among these, 40 were assessed for study eligibility, 34 were eligible, and 26 were successfully enrolled in the study. The enrollment efficiency ratio was 15.9% (26/164). The total amount spent on the advertisements was $2447.69. The cost per enrollment was $94.14.

It is important to note that when the paid advertisements were first launched, all three advertisements (including the one in Spanish) directed interested individuals to a contact form hosted on the University of Rochester (UR) Clinical and Translational Science Institute (CTSI) website. Our team noticed that, in the first few weeks, the advertisements were only resulting in completed contact forms and the recruitment of English-speaking participants. Since the UR CTSI website was solely available in English, we created a contact form in Spanish hosted in REDCap and directed the advertisements in Spanish to this form. After providing the contact form in Spanish, the advertisements resulted in completed contact forms and the recruitment of Spanish-speaking participants.

Another important point to note is that our team became aware of potentially fraudulent contact form submissions. For example, four contact forms (2.4%, 4/164) included different names but identical phone numbers. Moreover, 59 contact forms (36.0%, 59/164) included phone numbers that were connected through a Voice over Internet Protocol (VoIP; e.g. the call would start with ‘Hello! Please state your name after the tone and Google Voice will try to connect you’). VoIP numbers can be used in fraudulent activities given their ability to choose any calling area code (e.g. 913 for Kansas City, 585 for Western New York), even if originating from an international location (e.g. someone based in India can use a VoIP number with a US area code)^13^. Conducting eligibility over the phone and not directly online facilitated the verification of interested individuals. Moreover, utilizing a mailed incentive helped identify potentially fraudulent participation from outside the US.

The participants’ mean age was 22.7 years (SD=1.6). Half of the participants (50%) were male, 38.5% were female, and 11.5% were gender non-conforming/non-binary. Two-thirds of participants (69.2%) were born in the US, 23.1% in Puerto Rico, and 7.7% in Mexico. Eight participants (30.7%) selected Spanish as their language of preference. In terms of the type of vaping device, 16 participants (61.5%) indicated using disposables, 6 (23.1%) cartridges/pods, and 4 (15.4%) tanks/refillable. Sixteen participants (61.5%) reported using marijuana in e-cigarettes. Six participants (23.1%) had high e-cigarette dependence. Twenty participants (76.9%) had attempted to quit e-cigarettes in the past year (Table 1).

**Table 1:** Baseline characteristics of US Latino young adults (aged 18–25 years) enrolled in a vaping cessation study via social media, 2023 (N=26)

*Characteristics*	*n (%)*
**Age** (years), mean (SD)	22.7 (1.7)
**Gender**	
Female	13 (50.0)
Male	10 (38.5)
Gender variant/non-conforming/non-binary	3 (11.5)
**Sexual orientation**	
Heterosexual or straight	12 (46.2)
Homosexual or gay	5 (19.2)
Bisexual	7 (26.9)
Pansexual	1 (3.8)
I am not sure/questioning	1 (3.8)
**Marital status**	
Married/cohabitating	3 (11.5)
Single	23 (88.5)
**Education level**	
High school or equivalent (12th grade)	12 (46.2)
Associate’s degree (2-year college)	2 (7.6)
Technical school	3 (11.5)
Bachelor’s degree (4-year college)	6 (23.0)
Graduate degree (Master’s or Doctorate)	3 (11.5)
**Employment status^a^**	
Employed	13 (50.0)
Unemployed	5 (19.2)
Homemaker	1 (3.8)
Student	9 (34.6)
**Country of birth**	
Mexico	2 (7.7)
Puerto Rico	6 (23.1)
USA	18 (69.2)
**Language**	
Only Spanish	1 (3.8)
More Spanish than English	3 (11.5)
Both equally	9 (34.6)
More English than Spanish	6 (23.1)
Only English	7 (26.9)
**Type of device**	
Cartridge/pod device	6 (23.1)
Disposable	16 (61.5)
Tank/refillable	4 (15.4)
**Quit attempt in the past year**	
Yes	20 (76.9)
No	6 (23.1)
**Use of marijuana in e-cigarettes**	
Yes	16 (61.5)
No	10 (38.5)
**Electronic cigarette dependence^b^**	
Not dependent	1 (3.8)
Low	11 (42.3)
Medium	8 (30.8)
High	6 (23.1)

a The total is not 26 because the participants could have had more than one employment status.

b E-cigarette dependence was measured by the Penn State E-cigarette Dependence Index (PSECDI); PSECDI scores: 0–3 ‘Not dependent’, 4–8 ‘Low dependence’, 9–12 ‘Medium dependence’, and ≥13 ‘High dependence’.

## DISCUSSION

To the best of our knowledge, this is the first study assessing the feasibility and cost of recruiting Latino young adults into a vaping cessation study via social media. This work demonstrates that social media is a feasible and relatively low-cost approach to recruiting Latino young adults who vape. Moreover, it resulted in the recruitment of Latinos with a range of sociodemographic and vaping-related characteristics.

The cost per enrollment was $94.14. This result is appropriate as it falls within the range of previously reported recruitment costs for Latinos using social media. In 2015, Chalela et al.^14^ implemented a social media campaign on Facebook to promote Quitxt, a smoking cessation text messaging program, among Latino young adults (aged 18–29 years)^14^. The campaign resulted in a cost of $120 per enrollment in Quitxt^14^. Moreover, in 2016, Medina-Ramirez et al.^15^ implemented a social media campaign on Facebook to promote a smoking cessation RCT testing the efficacy of culturally relevant self-help booklets among Latinos^15^. The campaign resulted in a cost of $74.12 per enrollment in the RCT^15^.

The findings regarding the impact of providing the contact form in Spanish reinforce the importance of linguistically appropriate recruitment materials among Spanish-speaking Latinos^16^. Study teams should continually monitor enrolled participants’ overall recruitment numbers and baseline characteristics to ensure they resemble the study population.

The presence of potentially fraudulent contact form submissions is consistent with previous studies conducting recruitment via social media^17^. As done in this study, conducting eligibility over the phone and utilizing a mailed incentive assisted in verifying eligible individuals.

### Limitations

Some limitations should be considered when interpreting the findings. First, this study was not designed to test recruitment efficiency via social media. Second, participants were solely recruited via Facebook and Instagram. Future studies should also recruit young Latino adults via different social media platforms [e.g. Snapchat, TikTok, X (formerly known as Twitter)]. Third, the advertisements were designed by the research team, not a professional marketing team. Future research could benefit from the contributions of social media experts. Lastly, the cost analysis only included the costs related to launching the paid advertisements and not the time spent designing the advertisements, contacting interested individuals, or participant incentives.

## CONCLUSIONS

It is feasible to recruit Latino young adults into a vaping cessation study via social media. Social media offers a relatively low-cost approach to recruiting a diverse sample of Latino young adults who vape.

## Data Availability

The data supporting this research are available from the authors on reasonable request.
